# Putting patient value first: Using a modified nominal group technique for the implementation of enhanced recovery after cardiac surgery recommendations

**DOI:** 10.1016/j.xjon.2022.07.004

**Published:** 2022-07-20

**Authors:** Mudra G. Dave, Anna M. Chudyk, Nebojša Oravec, David E. Kent, Todd A. Duhamel, Annette S.H. Schultz, Rakesh C. Arora

**Affiliations:** a Faculty of Kinesiology and Recreation Management, University of Manitoba, Winnipeg, Manitoba, Canada; b Cardiac Sciences Manitoba, St Boniface Hospital, Winnipeg, Manitoba, Canada; c St Boniface Research Centre, Health Services & Structural Determinants of Health Research, Winnipeg, Manitoba, Canada; d College of Nursing, Rady Faculty of Health Sciences, University of Manitoba, Winnipeg, Manitoba, Canada; e Max Rady College of Medicine, University of Manitoba, Winnipeg, Manitoba, Canada; f Institute of Cardiovascular Sciences, Alberchtsen Research Centre, St Boniface Hospital, Winnipeg, Manitoba, Canada; g Section of Cardiac Surgery, Department of Surgery, Rady Faculty of Health Sciences, University of Manitoba, Winnipeg, Manitoba, Canada

**Keywords:** cardiac surgery, perioperative guidelines, nominal group technique, patient-centered, enhanced recovery protocols, ERAS-CS, Enhanced Recovery After Surgery–Cardiac Surgery, NGT, nominal group technique, PONV, postoperative nausea and vomiting, Q1, first quartile, SSI, surgical site infection

## Abstract

**Objective:**

In 2019, the Society for Enhanced Recovery After Cardiac Surgery (ERAS-CS) published perioperative guidelines to optimize the care of patients undergoing cardiac surgery. For centers with limited capacity, a sequential approach to the implementation of the full guidelines may be more feasible. Therefore, we aimed to explore the priority of implementation of the ERAS-CS guideline recommendations from a patient and caregiver perspective.

**Methods:**

Using a modified nominal group technique, individuals who previously underwent cardiac surgery and their caregivers ranked ERAS-CS recommendations within 3 time points (ie, preoperative, intraoperative, and postoperative) and across 2 to 3 voting rounds. Final round rankings (median, mean and first quartile) were used to determine relative priorities.

**Results:**

Seven individuals (5 patients and 2 caregivers) participated in the study. Patient engagement tools (2, 2.29, and 1.50), surgical site infection reduction (2, 1.67, and 1.25), and postoperative systematic delirium screening (1, 2.43, and 1.00) were the top-ranked ERAS-CS recommendations in the preoperative, intraoperative, and postoperative time points, respectively.

**Conclusions:**

Exploration of patient and caregiver priorities may provide important insights to guide the healthcare team with clinical pathway development and implementation. Further study is needed to understand the impact of the integration of patient and caregiver values on effective and sustainable clinical pathway implementation.

## Graphical abstract

**Figure:**
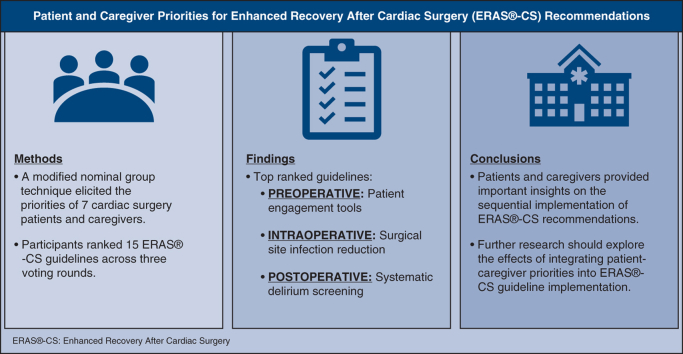

**Figure:**
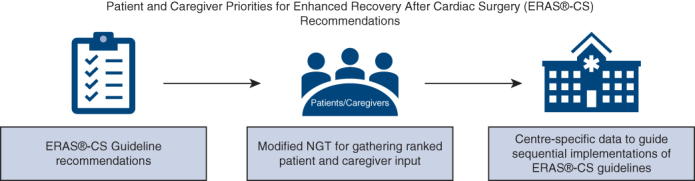
Exploration of patient and caregiver priorities for ERAS-CS recommendations.

**Central Message**

Group consensus techniques have the potential to elicit important patient and caregiver insights that may help the healthcare team with clinical pathway development and implementation.

**Perspective**

Enhanced Recovery After Cardiac Surgery (ERAS-CS) guidelines were recently developed to optimize patient recovery. However, not all centers have the resources to implement the guideline recommendations simultaneously. This study used a patient and caregiver group consensus technique that can be used to explore priorities to guide the sequential implementation of ERAS-CS guideline recommendations.

The proportion of people classified as older adults has been increasing annually in Canada, with a projected 60 million Canadians aged ≥65 years by the year 2031.^1^ Consequently, more older adults with cardiovascular disease who have higher rates of comorbidities, such as frailty, are requiring surgical intervention.^2^ Traditionally, cardiac surgery–related outcomes research has focused largely on achieving success as it pertains to metrics such as intensive care unit length of stay, readmission rates, and mortality rates.^3^, ^4^, ^5^ More recently, there has been a concerted shift in focus towards multifaceted clinical strategies to ensure that older patients not only survive, but also thrive after cardiac surgery.^6^

To this end, the Enhanced Recovery After Cardiac Surgery (ERAS-CS) has provided a bundle of 22 recommendations that endorse a multimodal and interdisciplinary approach to cardiac surgery perioperative care (collectively referred to as ERAS-CS guidelines). These recommendations aimed to improve clinical and patient-centered outcomes through evidence-based care.^6^ Early evidence supports the positive impact of ERAS-CS guidelines on patient outcomes.^7^^,^^8^ However, in previous reports, full ERAS-CS bundle implementation primarily occurred at sites with sufficient capacity to do so, which might not be possible in many resource-limited centers. In such centers, a sequential approach to the implementation of the full guidelines may be more feasible, but the prioritization or sequence remains unclear. Furthermore, incorporation of patients and caregivers (ie, nonclinical persons with interest in the patient's well-being who are not remunerated for their role in the patient's life^9^) offer unique and important perspectives that should be considered when prioritizing the sequential implementation of ERAS-CS guidelines, as they are deeply embedded in the cardiac surgical care receiving process. As the receivers (rather than providers) of cardiac surgery care, patients and caregivers provide the healthcare team with a unique perspective in understanding the perceived importance of change. Although there is evidence to support the positive impacts of engaging patients and caregivers in shaping health care (and health research) on individual health and clinical outcomes,^10^ at this point there is limited knowledge of patient and caregiver perspectives on the priority of the individual recommendations that compose the ERAS-CS guidelines.^11^^,^^12^

The aim of this exploratory study was to gather the perspectives of previous cardiac surgery patients and caregivers on the relative importance of the ERAS-CS guideline recommendations within each of the time points (ie, preoperative, intraoperative, and postoperative) of the patients' surgical journey using a modified nominal group technique (NGT). These findings will provide preliminary data on patient and caregiver perspectives on the relative importance of select ERAS-CS guideline recommendations and model how other centers can use an established consensus technique to gather patient and caregiver perspectives on the relative priorities of quality improvement initiatives (Figure E1).

## Methods

### Study Design

This study used a modified NGT to engage patients who had received a cardiac surgical intervention and their caregivers in prioritizing and discussing ERAS-CS recommendations at the St Boniface Hospital. At the time of the study, the study hospital did not have an ERAS-CS program and was developing a plan for the sequential implementation of the full ERAS-CS guidelines. Ethics approval for this study was granted by the University of Manitoba's Health Research Ethics Board (HS23729; H2020:126) and St Boniface Hospital's Research Review Committee (2020/1909) on May 11, 2020. All individuals provided written informed consent prior to study participation. Reporting of the study results was guided by the consensus group method reporting guidelines.^12^ NGT proceedings were recorded by a notetaker.

In line with standard NGT practice, our targeted sample size was 5 to 12 participants.^13^^,^^14^ This recommended sample size best supports the purpose of adopting the NGT, which is to gather a balanced variety of perspectives in a face-to-face interaction.^15^^,^^16^ Inclusion criteria were limited to patients who had previously undergone cardiac surgery within the last year at the study hospital and consented to be listed in a database of individuals interested in future research and their caregivers. Exclusion criteria were an inability to speak, read, or write in the English language and an inability to provide written informed consent. A purposeful convenient sampling method was used to recruit participants by phone with the aim of capturing equal sex-based representation. Caregivers' contact information was obtained from successfully contacted patients.

The NGT is a consensus method that promotes person-to-person discussion, takes relatively little time to conduct, and provides immediate results.^14^^,^^15^ Although the NGT is underused in adult cardiac surgical research, it is a well-established group consensus method in the wider literature; to illustrate, a search of the technique in PubMed yielded almost 200 articles published in the first half of 2022. In contrast to the NGT, the Delphi technique, arguably a more commonly known consensus method, is typically conducted outside of a group setting, requires larger groups of “experts,” takes longer to complete, and relies on electronic or mailed-in self-reported questionnaires.^14^ Previous studies have recommended using the Delphi technique if the aim of the study involves developing guidelines,^14^ which is not in line with the aim of the present study. In addition, because the Delphi technique is typically being conducted outside of a group setting, it has been described as being a “complex method for lay people,” as there are no clarification or discussion steps involved in the design.^14^ Unlike a focus group, the NGT aims to reach consensus and is structured, which prevents individuals or subgroups from dominating the discussion.^14^^,^^15^ Although focus groups allow for an in-depth discussion of a research question, they do not aim to arrive at structured group decision making, as is possible through consensus methods.^14^ These key differences in the Delphi technique, focus groups, and the NGT helped our research team identify the NGT as a more suitable consensus method for our study.

**Figure 1:**
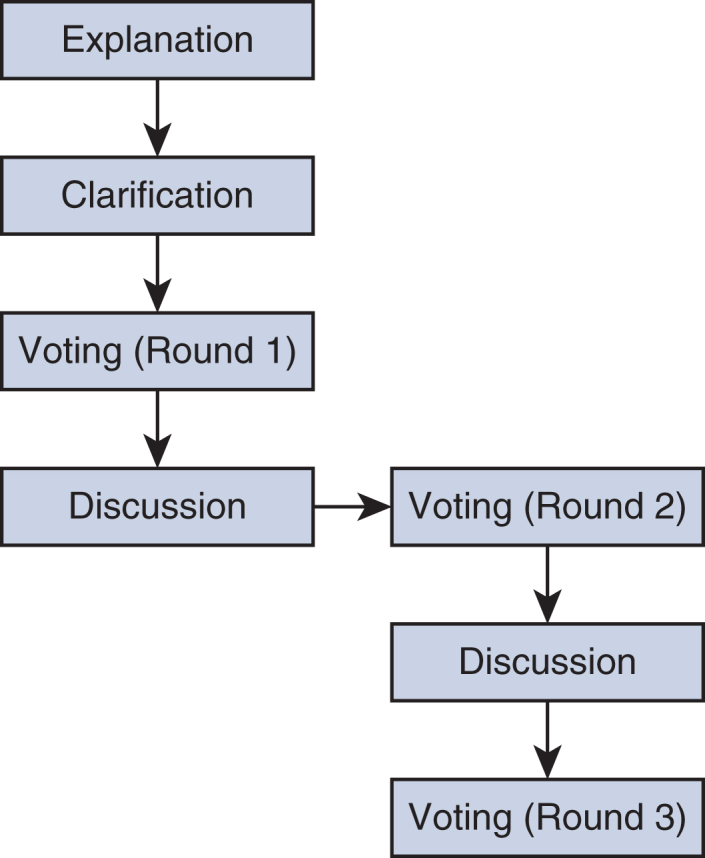
Study design: modified nominal group technique process.

A typical NGT consists of 4 stages: (1) silent generation (of ideas), (2) round-robin (sharing of ideas), (3) clarification and discussion (of ideas), and (4) voting (ranking).^14^, ^15^, ^16^, ^17^ The last 3 stages are typically repeated twice, working toward reaching an agreement among participants. The NGT process often has been described as a flexible consensus method that can be modified to fit the availability of literature on a study's topic, participant's voice, and study's purpose and aim of generalizability.^14^, ^15^, ^16^, ^17^, ^18^ In this study, the silent generation of ideas stage was replaced with an explanation stage as participants voted on a priori identified recommendations found in the literature^14^ (Figure 1). Specifically, prior to data collection, a list of 14 ERAS-CS recommendations was developed for ranking. These recommendations were not chosen in an attempt to create a “new” or “an abbreviated” version of the full ERAS-CS guidelines. Indeed, to gain the potential benefits, the guidelines intend for all the recommendations to be implemented, because the individual importance of each recommendation is unclear. Rather, the recommendations were chosen based on their direct relevance to patients and caregivers. Moreover, the remaining 8 ERAS-CS recommendations were not included because they had been either already implemented or considered by our center as standard of practice that would need to be implemented into our current daily clinical practice. In addition, a “diet and bowel regimen and postoperative nausea and vomiting (PONV)” recommendation was also included in the voting list based on its frequent occurrence in postoperative cardiac surgery patients as well as the emerging ERAS-CS literature.^7^^,^^19^^,^^20^ Thus, participants voted on a total of 15 recommendations, organized into 3 time points: 6 preoperative, 2 intraoperative, and 7 postoperative (Table 1). Prior to data collection, participants received a document that provided an overview of the recommendations to be ranked.

**Table 1:** Overview of the 15 ERAS-CS recommendations ranked by study participants^6^^,^^7^

Time point	ERAS-CS recommendation	Description
Preoperative	Preoperative correction of nutritional deficiency	Providing oral nutritional supplementation preoperatively for patients deemed at risk for malnutrition
	Consumption of clear liquids before general anesthesia	Encouraging clear liquids 2 h preoperatively before general anesthesia has been shown to be safe.
	Preoperative carbohydrate loading	A complex carbohydrate beverage 2 h preoperatively has shown to improve postoperative outcomes such as improved glucose control and return of gastrointestinal function.
	Patient engagement tools	Preoperative education and counseling tools may help reduce perioperative fear, fatigue, and discomfort.
	Prehabilitation	Preoperative rehabilitation has been shown to enable patients to withstand the stress of surgery by improving functional capacity and insulin sensitivity and reducing postoperative complications.
	Smoking and hazardous alcohol consumption	Preoperative alcohol and smoking use have been shown to have negative postoperative outcomes such as delirium and respiratory complications.
Intraoperative	Surgical site infection reduction	A surgical site infection care bundle consisting of topical intranasal therapy, intravenous cephalosporin prophylactic antibiotic, clipping of hair as opposed to shaving, and using chlorhexidine-alcohol based solution for skin preparation before surgery may reduce postoperative surgical site infections.
	Rigid sternal fixation	Rigid plate fixation resulted in reduction of pain and improved upper-extremity function and quality-of-life scores compared with standard wire cerclage.
Postoperative	Perioperative glycemic control	Perioperative glycemic control–based interventions have been shown to help reduce postoperative glucose levels and surgical site infection.
	Pain management	The use of postoperative opioids for pain management has been shown to decrease with the use of acetaminophen. Preoperative counseling can potentially help set pain-related expectations with patients and decrease opioid use.
	Postoperative systematic delirium screening	Postoperative delirium screening has been shown to help with early identification and prompt the healthcare team to seek potential underlying causes, translating to a decrease in long-term negative consequences.
	Chest tube patency	Maintaining chest tube patency without “stripping” or breaking the sterile field to remove blood clots is recommended.
	Kidney stress and acute kidney injury	Early identification of kidney stress using biomarkers may help prevent postoperative acute kidney injury.
	Goal-directed fluid therapy	Goal-directed fluid therapy has been shown to improve postoperative outcomes and hospital length of stay.
	Diet and bowel regimen and postoperative nausea and vomiting∗	The use of antinausea (antiemetics) and prokinetic agents are recommended for postoperative gastrointestinal issues.

*ERAS-CS*, Enhanced Recovery After Cardiac Surgery.

∗ Diet and bowel regimen and postoperative nausea and vomiting is not part of the ERAS-CS guidelines^6^; it was included based on the study by Williams and colleagues (2018).^7^

The explanation stage was included at the start of the NGT session. A cardiac surgeon research team member (R.C.A.) provided the participants with an overview presentation explaining the ERAS-CS guidelines. Participants were then provided with the opportunity to ask clarifying questions and engage in related discussions before the voting stage. Participants' clarifying questions were answered by 2 clinician research team members (R.C.A. and A.S.H.S.). Participants then anonymously ranked the perceived importance (ie, priority) of the recommendations within each time point on their private ranking sheets using a Likert scale. Once the participants finished voting, a study team member (D.E.K.) tallied the results and shared the aggregated rankings (by time point) with the participants. Open discussion aimed at reaching consensus was then encouraged before another round of voting. The total number of voting rounds was determined a priori to be 3 or fewer if an agreement (ie, consensus) was reached.^14^ At this stage, agreement was defined as the point at which all participants did not change their rankings (ie, nonvarying responses) between consecutive voting rounds.

### Data Analysis

Participants' descriptive characteristics were summarized using count (percentage) and median (interquartile range). For each voting round, rankings within each time point were calculated using median, mean, and first quartile (Q1) values. Final round rankings were used to determine the overall rankings (ie, perceived within-group priority of the recommendations). The median was used primarily to determine within-group ranking. In the event that the median within-group rank was the same for 2 or more recommendations, the mean was used to determine relative priority. A lower mean represented greater priority and a higher rank. If the median and mean were the same, the Q1 value was used to determine the rankings. Similarly, in this situation, a lower Q1 value represented greater priority and a higher rank. Finally, once data analyses were completed, the research team went through the notetaker notes to identify key quotes that illustrated participants' reasoning behind the top and lowest final rankings. Data analyses were conducted using Microsoft Excel version 16.52.

## Results

A total of 12 patients were contacted, of whom 7 and 2 of their caregivers expressed interest in study participation. However, 2 of these patients were unable to complete the data collection step for personal reasons. Thus, 5 patients and 2 caregivers provided written informed consent and participated in the study. Three patients (60%) and 1 caregiver (50%) were female. The median age of the patients and caregivers was 66 years and 74.5 years, respectively. Selected sociodemographic characteristics of study participants are provided in Table 2. All participants voted in each of the ranking rounds for the preoperative and postoperative recommendations. One of the patients did not rank the intraoperative recommendations in round 2 because they took a personal phone call.

**Table 2:** Demographic and clinical characteristics of study participants

Characteristic	Value
Patient characteristics (n = 5)	
Demographics	
Age, y, median (IQR)	66 (9)
Age ≥65 y, n (%)	5 (100)
Female sex, n (%)	3 (60)
Male sex, n (%)	2 (40)
Procedure type, n (%)	
Isolated coronary artery bypass grafting	3 (60)
Isolated valve	2 (40)
Hospital length of stay, d, median (IQR)	
Intensive care unit stay	1 (2)
Recovery ward stay	5 (3)
Patient type, n (%)	
Elective	5 (100)
Caregiver characteristics	
Demographics	
Age, y, median (IQR)	74.5 (5.5)
Age ≥65 y, n (%)	2 (100)
Female sex, n (%)	1 (50)
Male sex, n (%)	1 (50)
Relationship to patient, n (%)	
Spouse	2 (100)

*IQR*, Interquartile range; *y*, years; *d*, days.

### Changes in Rank Order Across Voting Rounds

Participants' rankings changed between the first and third voting rounds for select preoperative and postoperative recommendations, whereas the rank order of the intraoperative recommendations remained the same between voting rounds (Table 3). Specifically, for the preoperative time point, preoperative correction of nutritional deficiency moved from position 2 to position 3, patient engagement tools moved from position 3 to position 1, and prehabilitation moved from position 1 to position 2. The rank order of the other 3 recommendations remained the same. For the postoperative time point, 5 of the 7 recommendations changed between rounds 1 and 3. Perioperative glycemic control changed from position 6 to position 4, chest tube patency changed from position 5 to position 7, kidney stress and acute kidney injury changed from position 3 to position 5, goal-directed fluid therapy changed from position 4 to position 3, and diet and bowel regimen and PONV changed from position 7 to position 6. The top-2 ranked postoperative recommendations (ie, postoperative systematic delirium screening and pain management) did not change positions between rounds 1 to 3. Individual participant rankings also changed between the first and final voting rounds. Specifically, 6 of the 7 participants (86%) changed their rank ordering of preoperative and postoperative recommendations, and 4 of the 7 (57%) changed their rank ordering of the intraoperative recommendations.

**Table 3:** Ranking of the 15 recommendations, by time point

Time point	ERAS-CS recommendation	Round 1, median (mean, Q1)	Round 2, median (mean, Q1)	Round 3, median (mean, Q1)
Preoperative	Preoperative correction of nutritional deficiency	2 (2.29, 2.00)	3 (3.17, 3.00)	3 (3.00, 3.00)
	Consumption of clear liquids before general anesthesia	4 (3.71, 3.00)	5 (4.50, 4.25)	5 (4.29, 3.00)
	Preoperative carbohydrate loading	5 (4.57, 4.00)	6 (5.00, 4.25)	5 (4.29, 3.50)
	Patient engagement tools	2 (2.71, 1.50)	1 (2.33, 1.00)	2 (2.29, 1.50)
	Prehabilitation	1 (2.29, 1.00)	2 (2.83, 1.00)	2 (2.71, 1.50)
	Smoking and hazardous alcohol consumption	4 (3.29, 1.00)	3 (3.17, 2.50)	3 (3.43, 2.50)
Intraoperative	Surgical site infection reduction	2 (1.57, 1.00)	2 (1.67, 1.25)	
	Rigid sternal fixation	2 (1.86, 2.00)	2 (2.00, 2.00)	
Postoperative	Perioperative glycemic control	5 (5.00, 4.00)	5 (4.57, 3.50)	4 (4.00, 3.00)
	Pain/discomfort management	2 (2.71, 1.50)	2 (2.14, 1.50)	2 (2.43, 2.00)
	Postoperative systematic delirium screening	1 (3.14, 1.00)	2 (3.14, 1.00)	1 (2.43, 1.00)
	Chest tube patency	5 (4.29, 2.50)	6 (5.43, 4.50)	6 (6.14, 5.50)
	Kidney stress and acute kidney injury	4 (3.71, 3.00)	3 (3.57, 3.00)	4 (4.00, 3.50)
	Goal-directed fluid therapy	4 (3.86, 3.00)	4 (4.00, 3.50)	3 (3.43, 3.00)
	Diet and bowel regimen and postoperative nausea and vomiting	5 (5.29, 4.00)	5 (5.14, 4.50)	6 (5.57, 5.00)

*ERAS-CS*, Enhanced Recovery After Cardiac Surgery.

### Final Rankings by Time Point

**Figure 2:**
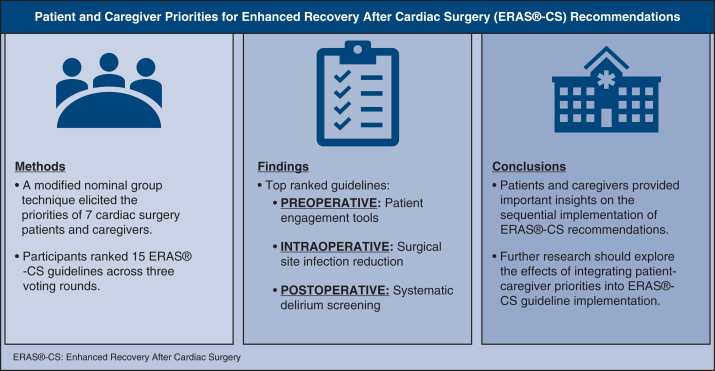
Visual summary of methods, results, and implications of the investigation of patient and caregiver priorities for Enhanced Recovery After Cardiac Surgery (*ERAS-CS*) recommendations.

As shown in Table 4, patient engagement tools, surgical site infection (SSI) reduction, and postoperative systematic delirium screening were the final top-ranked recommendations in the preoperative, intraoperative, and postoperative time points, respectively. Key quotes illustrating participants' reasoning behind the top-ranked and lowest-ranked ERAS-CS recommendations are presented in Tables 5 and 6, respectively. Note that in the preoperative time point, 2 recommendations (ie, consumption of clear liquids before general anesthesia and preoperative carbohydrate loading) had the same median (5.00) and mean (4.29) values; therefore, Q1 was used to determine the final ranking. Similarly, in the postoperative time point, 2 recommendations (ie, preoperative glycemic control and kidney stress and acute kidney injury) had the same median (4.00) and mean (4.00) values, and hence Q1 was used to determine the final ranking. All other ties were resolved using means. The lowest-ranked recommendations were preoperative carbohydrate loading, rigid sternal fixation, and diet and bowel regimen and PONV in the preoperative, intraoperative, and postoperative time points, respectively. A summary of the results is provided in Figure 2.

**Table 4:** Consensus rankings of the 15 recommendations, by time point

Time point	ERAS-CS recommendation	Ranking
Preoperative	Preoperative correction of nutritional deficiency	3
	Consumption of clear liquids before general anesthesia	5
	Preoperative carbohydrate loading	6
	Patient engagement tools	1
	Prehabilitation	2
	Smoking and hazardous alcohol consumption	4
Intraoperative	Surgical site infection reduction	1
	Rigid sternal fixation	2
Postoperative	Perioperative glycemic control	4
	Pain/discomfort management	2
	Postoperative systematic delirium screening	1
	Chest tube patency	7
	Kidney stress and acute kidney injury	5
	Goal-directed fluid therapy	3
	Diet and bowel regimen and postoperative nausea and vomiting	6

*ERAS-CS*, Enhanced Recovery After Cardiac Surgery.

**Table 5:** Key quotes from patients and caregivers for the top-ranked ERAS-CS recommendations

ERAS-CS recommendations	Key quotes
Patient engagement tools	This idea of how do you present information in a meaningful way that people are open to receiving and come forward to ask questions. That perspective was a really interesting one for me to hear, because when I'm one on one [alone with the physician at the appointment], I do the same thing [not able to ask questions], I try to get out early.
	If you just hand someone reading material, don't count on that being very helpful to a lot of people. Everyone might read it, but they might have trouble understanding it.
Surgical site infection reduction	I would think you would have much more pain if you have an infection, so you would have to really have to care for the surgical site and care for the wound initially, and then the pain management would come after in my view, because that's connected with the surgery.
	You get bathing stuff before surgery and then when you are in the hospital, they are hyper-focused on infectious control.
Postoperative delirium screening	I think everyone experiences it differently. You don't know you have it, but you know something is off. You're thinking, where is this, what's happening. For me, while I was in it, I thought whatever; I'm just not feeling well. However, I think for the caregivers, it's important because it's on our behalf.
	The physical health things seem to be more easily dealt with because there is medication or whatever, whereas with delirium it's different for everybody. The length of time you have it is different, how you progress pass it is also different. It's very scary. It's the unknown.

*ERAS-CS*, Enhanced Recovery After Cardiac Surgery.

**Table 6:** Key quotes from patients and caregivers for the lowest-ranked ERAS-CS recommendations

ERAS-CS recommendation	Key quotes
Preoperative carbohydrate loading	I think at the time [of surgery], my least concern is what I consume prior to surgery, as there were many more life-and-death questions I had at the time. In retrospect, this could be a question that may get more consideration after the life crisis has been dealt with.
Rigid sternal fixation	I'm in a quandary about rigid sternal fixation. I still marked it as #3, but if more studies show that's the way to go then, yes, I think that would be the way to go, but I don't know enough about it. That is in [the surgeon's] wheelhouse, but if it is found to be the best in terms of reducing pain, then yes.
Diet and bowel regimen and postoperative nausea and vomiting	They [healthcare staff] give you a laxative right away.

*ERAS-CS*, Enhanced Recovery After Cardiac Surgery.

## Discussion

This study used a modified NGT to identify the relative priority of select ERAS-CS recommendations from the perspectives of cardiac surgery patients and their caregivers. Final rankings identified patient engagement tools, SSI reduction, and postoperative systematic delirium screening as the highest ranked patient and caregiver recommendations in the preoperative, intraoperative, and postoperative time points, respectively. To our knowledge, this is one of the first studies to explore patient and caregiver perspectives on the relative priority of ERAS-CS guidelines.

Patient engagement tools was ranked as a top priority. Participants articulated that the manner in which information about their perioperative journey is shared with them and their loved ones is key to patient engagement. This is supported by the literature, which reports strong information-related preferences among cardiac surgery patients and caregivers^9^ and indicates that inadequate information delivery may contribute to misunderstanding, forgetting, or ignoring important healthcare information, difficulties with compliance and adherence to recommended treatment strategies, and poor health outcomes.^21^^,^^22^ Mobile health applications show great promise as patient engagement tools and have been associated with increased patient satisfaction and decreased periprocedural anxiety, length of stay, and 30-day, 60-day, and 90-day inpatient readmissions in surgical and nonsurgical populations.^23^^,^^24^ Centers implementing ERAS-CS guidelines should look to the small but emerging body of literature surrounding mobile health applications for perioperative cardiac surgery that best support their local contexts.^24^^,^^25^

Among the intraoperative enhanced recovery protocols, SSI reduction was identified as the top patient and caregiver valued priority. Participants relayed the importance of SSI reduction to minimize the possibility of worse postoperative pain. SSIs are preventable complications, observed to occur at a rate of 1.3% to 12.8% following cardiac surgery and associated with increased mortality in patients and financial burden on the healthcare system.^26^, ^27^, ^28^ Previous research suggested decreased patient-reported outcomes (ie, physical health, global mental health scores, and physical functioning) in patients with deep sternal wound infections, prolonged ventilation, stroke, and renal failure.^29^ SSIs remain a significant burden for cardiac surgery patients and their caregivers and a valued component of enhanced recovery protocols.

Systematic delirium screening was recognized as a top priority within the postoperative ERAS-CS guidelines. Delirium is an acute neurocognitive disorder that has been observed in 26% to 52% of all cardiac surgery patients.^30^^,^^31^ Delirium often leads to worse postoperative outcomes, such as prolonged hospital and intensive care unit length of stay and an elevated risk of mortality.^32^^,^^33^ Furthermore, patient-reported experiences of delirium often have been described as frightening and causing distress to the patient and their caregiver similar to our study's findings (Table 5).^34^ As such, the continued use of delirium screening tools and refinement of delirium management received advocacy as an important area of focus for the perioperative team implementing enhanced recovery protocols.

Preoperative carbohydrate loading, rigid sternal fixation, and diet and bowel and PONV were the lowest ranked priorities across the 3 time points by patients and caregivers. The low rank of preoperative carbohydrate loading in the study was attributed to the focus on factors perceived to be most closely related to postoperative survival at this time point. Rigid sternal fixation was ranked as the lowest priority during the intraoperative time point, as the participants felt that they had inadequate clinical knowledge to understand the importance of different sternal fixation methods. The low ranking of PONV was unexpected, as it is a common post–cardiac surgery outcome, nausea occurring in 15.3% and vomiting in 10.3% of patients causing distress and discomfort postoperatively.^19^^,^^20^ This discrepancy between the published literature and the results of the present study can be hypothesized to be related to our study participants' lack of personal experience with PONV.^20^

### Limitations

This study has some limitations that should be noted. First, the recruitment of both patients and caregivers occurred at only a single tertiary medical center, which might have an impact on the generalizability of the results to other clinical settings. Moreover, the relatively small number of participants typically recommended for NGTs precludes us from being able to claim that our findings are representative of all patients and caregivers undergoing cardiac surgery within or outside the study hospital. However, the intent of this investigation was not to provide a universal road map, but rather to demonstrate the utility of patient and caregiver involvement in helping inform where our local team should focus their initial efforts in the sequential implementation of the full ERAS-CS guidelines. Centers with diverse populations or more resources could consider holding multiple NGT groups and referring to articles by McMillan and colleagues^16^ or Van Brenda^35^ for guidance on navigating the complexity of analyzing data across multiple NGT groups. A second limitation warranting mention is that participants were asked to recall their cardiac surgery journey from upward of 1 year after their procedure date, potentially introducing some degree of recall bias. Third, participants' opinions were elicited while they were in the same room as one another, which might have introduced pressure to conform in a group setting. We aimed to minimize this bias by using an established group consensus technique that balanced group discussions with an anonymous ranking of recommendations.

## Conclusions

The roles of patients and caregivers within both health practice and research are changing. Patient-caregiver voices recorded through consensus techniques such as the NGT can help healthcare professionals (eg, cardiac surgeons) and policy makers guide the sequential implementation of a multicomponent care bundle such as the ERAS-CS guidelines into clinical practice. Key patient and caregiver value priorities were identified, including patient engagement tools, SSI reduction, and postoperative systematic delirium screening. We suggest that future guideline development and implementation initiatives consider directly incorporating patient and caregiver perspectives, perhaps even across multiple groups of participants.^16^ Further research is needed to understand the impact of integrating patient and caregiver values on the effectiveness (eg, measuring patient satisfaction) and sustainability (eg, measuring hospital outcomes) of ERAS-CS pathway implementation.

### Conflict of Interest Statement

Dr Arora has received an unrestricted educational grant from 10.13039/100013739Pfizer Canada Inc and honoraria from Abbott Nutrition, Edwards Lifesciences, and AVIR Pharma for work unrelated to this study. All other authors reported no conflicts of interest.

The *Journal* policy requires editors and reviewers to disclose conflicts of interest and to decline handling or reviewing manuscripts for which they may have a conflict of interest. The editors and reviewers of this article have no conflicts of interest.

## Figures and Tables

**Figure E1:**
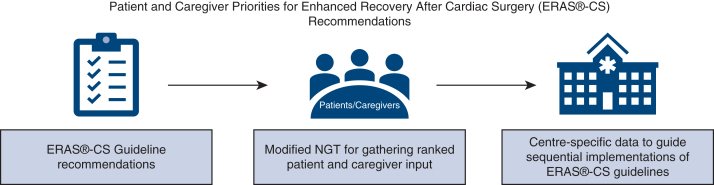
Snapshot of the study process. *ERAS-CS*, Enhanced Recovery After Cardiac Surgery; *NGT*, nominal group technique.
